# Emerging antibiotic resistance in *Vibrio cholerae*: a study of cholera prevalence and resistance patterns in Zambia’s Copperbelt Province

**DOI:** 10.1186/s12879-025-11259-w

**Published:** 2025-07-01

**Authors:** David Chisompola, John Nzobokela, Roy Moono, Elijah Chinyante, Allen Chipipa, Nancy Chapuswike, Moses Chakopo, Nswana Mukuma, Martin Chakulya

**Affiliations:** 1 Pathology Laboratory Department, Arthur Davison Children’s Hospital, Ndola, Zambia; 2 Pathology Laboratory Department, Kitwe Teaching Hospital, Kitwe, Zambia; 3 Pathology Laboratory Department, Ndola Teaching Hospital, Ndola, Zambia; 4 Pathology and Microbiology Department, Mulungushi University School of Medicine and Health Sciences, Livingstone, Zambia; 5 Pathology Laboratory Unit, Provincial Health Office, Ndola, Zambia

**Keywords:** Antimicrobial stewardship, Cholera, Multidrug resistance, *Vibrio cholerae*, Zambia

## Abstract

**Introduction:**

Cholera remains a significant public health challenge in Zambia, particularly in the Copperbelt Province, where antibiotic-resistant *Vibrio cholerae* strains are increasingly threatening treatment efficacy. This study aimed to determine the prevalence of cholera and the antibiotic resistance patterns of *V. cholerae* isolates at three tertiary hospitals in the region.

**Methods:**

A retrospective cross-sectional study was conducted across three major referral hospitals in the Copperbelt Province (Arthur Davison Children’s Hospital, Kitwe Teaching Hospital, and Ndola Teaching Hospital) during the cholera outbreak from January to April 2024. Clinical samples from suspected cholera cases were analysed, and antimicrobial susceptibility testing was performed following Clinical Laboratory Standards Institute guidelines and the European committee on antimicrobial susceptibility testing methodology for *Vibrio cholerae*. To isolate *Vibrio cholerae*, alkaline peptone water and thiosulfate-citrate-bile salt-sucrose agar were utilized. The isolates were identified based on colony morphology, Gram staining, biochemical testing, and serotyping. Antimicrobial susceptibility testing was conducted by using the Kirby-Bauer disk diffusion method. Descriptive statistics were employed to assess the prevalence of *Vibrio cholerae*, and chi-square tests were applied with *p*-values of < 0.05 indicating statistical significance.

**Results:**

Of the 892 suspected cases, 334 (37.4%) were confirmed as *V. cholerae* through culture. The highest number of *V. cholerae* confirmed cases was recorded at Ndola Teaching Hospital 221 (24.8%), followed by Kitwe Teaching Hospital 88 (9.9%), while Arthur Davison Children’s Hospital 25 (2.8%) reported the lowest. High antimicrobial resistance was observed trimethoprim/sulfamethoxazole 69 (74.2%), ampicillin 75 (54.3%), and imipenem 22 (46.8%). In contrast, erythromycin 25 (100%), gentamicin 6 (85.7%) and ciprofloxacin 118 (76.6%) remained highly effective. The overall prevalence of multidrug resistance (MDR) in *Vibrio cholerae* was 3.7%. Among these, resistance to four or more antibiotics was observed in 3 (1.2%), followed by resistance to the combination of Ciprofloxacin, Ceftazidime, and Tetracycline in 2 (0.8%). All other MDR patterns were detected in a single isolate each (0.4%).

**Conclusion:**

The high prevalence of antibiotic-resistant *Vibrio cholerae* in the Copperbelt Province underscores the urgent need for enhanced antimicrobial stewardship and robust surveillance systems to inform effective cholera control strategies. Sustainable public health impact can be achieved through targeted immunization campaigns in endemic areas combined with strengthened water, sanitation, and hygiene (WASH) interventions, including improved access to clean water, adequate sanitation infrastructure, hygiene promotion, and supportive policies, which are essential for reducing transmission and preventing future outbreaks in vulnerable populations. However, because the number of antibiotics used in antimicrobial susceptibility testing across isolates varies, these results should be interpreted cautiously. Such differences may affect the comparability and overall interpretation of resistance patterns.

## Background

Cholera is a severe diarrheal disease caused by the bacterium *Vibrio cholerae*, typically spread through contaminated water. It remains a significant public health challenge, particularly in regions with inadequate water, sanitation and hygiene (WASH) infrastructure [1]. The disease is typically spread through contaminated water and food, and its impact is most severe in low-resource settings where overcrowding and poor sanitation exacerbate transmission [2]. In 2023, sub-Saharan Africa experienced the 7th cholera pandemic, with reported cases rising to 535,321 and 4,007 deaths from 472,697 in 2022. Globally, cholera cases were reported in 45 countries in 2023, an increase from 44 in 2022 and 35 in 2021 [3]. While Africa saw a 125% surge in cases, the Middle East and Asia experienced a 32% decline [3]. Alarmingly, 60% of annual cholera-related deaths occur in sub-Saharan Africa, underscoring the region’s vulnerability to this disease [4].

Zambia, like many countries in sub-Saharan Africa, has faced recurrent cholera outbreaks since 1977, with endemic transmission in regions such as Lusaka, Luapula, and the Copperbelt Province [5]. The Copperbelt Province, a densely populated and rapidly urbanizing area, is particularly susceptible to cholera due to its inadequate sanitation infrastructure, limited access to clean water, and high population mobility [6]. The 2024 cholera outbreak in Zambia was the most severe to date, with 19,719 cases and 682 deaths reported nationwide [7]. However, localized data on cholera prevalence and antibiotic resistance patterns in the Copperbelt Province remain scarce, hindering the development of targeted public health strategies.

The global emergence of antimicrobial resistance (AMR) has further complicated cholera management, with *Vibrio cholerae* strains showing increasing resistance to commonly used antibiotics such as tetracycline, chloramphenicol, and ciprofloxacin [8]. This resistance threatens the effectiveness of current treatment protocols, particularly in resource-limited settings like Zambia, where access to alternative antibiotics is often constrained [9]. For the treatment of cholera, Doxycycline, Azithromycin, and Ciprofloxacin are recommended as first-line antibiotics [10]. Additionally, antimicrobial susceptibility testing has been recommended to be performed at a minimum for the following agents: Azithromycin, Ciprofloxacin, Nalidixic Acid, Tetracycline, and Trimethoprim/Sulfamethoxazole to ensure effective and targeted therapy [10]. While studies have documented varying resistance patterns in other regions of Zambia and neighbouring countries [11, 12], there is a critical lack of data on the antibiotic resistance profiles of *V. cholerae* strains circulating in the Copperbelt Province. This knowledge gap limits the ability to design effective treatment guidelines and underscores the need for localized surveillance.

This study aims to address these gaps by determining the prevalence of cholera and the antibiotic resistance patterns of *V. cholerae* strains in three major referral hospitals in the Copperbelt Province. By integrating epidemiological and microbiological data, this research seeks to provide critical insights into the local dynamics of cholera transmission and resistance, informing more effective public health interventions and treatment strategies. The findings of this study will contribute to the growing body of knowledge on AMR in *V. cholerae* and support efforts to combat cholera in Zambia and beyond.

## Materials and methods

### Study design, site and population

This was a hospital based, retrospective cross-sectional study conducted at three tertiary hospitals on the Copperbelt Province: Arthur Davison Children’s Hospital (ADCH), Kitwe Teaching Hospital (KTH), and Ndola Teaching Hospital (NTH). These hospitals were selected due to their high patient volume during the cholera outbreak and their International Organization for Standardization (ISO) 15189:2022-accredited laboratories, ensuring standardized diagnostic procedures. The study analysed clinical samples collected between January and April 2024, during the peak of the cholera outbreak in Zambia. The microbiology laboratories at all three study sites, NTH, ADCH, and KTH where samples were processed are housed within the main building of each respective hospital. This centralized setup facilitated standardized procedures for sample transportation, handling, processing, and data collection across all locations, ensuring consistency throughout the study.

**Fig. 1:**
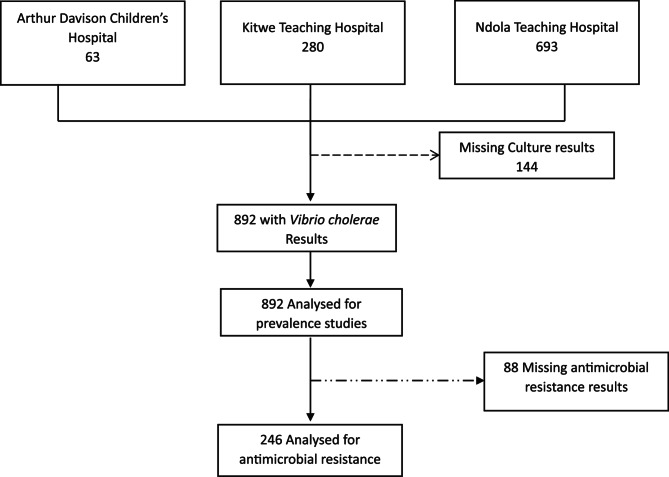
Participant selection

The study population comprised clinical samples submitted to the microbiology laboratories from patients with suspected cholera infection, characterized by acute watery diarrhoea. The sampling frame included all *Vibrio* species detected during the study period. Both inpatients and outpatients with recorded demographic data, including age, sex, stool culture results, and antimicrobial susceptibility test (AST) results, were included in the study. Samples missing key variables such as age, sex, stool culture results, or AST results were excluded from the analysis. A total of 892 suspected cholera cases were included, providing sufficient statistical power to detect significant trends in resistance patterns based on similar studies [11, 12]. The flow chart of study selection is presented in Fig. 1.

### Specimen processing

Samples were collected from suspected cholera cases, including rectal swabs placed in Cary-Blair transport medium and stool specimens collected in clean, sterile containers. All samples were promptly transported to the microbiology laboratory for processing and analysis [12]. A portion of each stool sample or rectal swab was directly inoculated and cultured onto Blood Agar (BA) (HiMedia Laboratories, Mumbai, India), MacConkey (MAC) (HiMedia Laboratories, Mumbai, India), and Thiosulfate-Citrate-Bile Salts-Sucrose (TCBS) (HiMedia Laboratories, Mumbai, India). The use of BA and MAC alongside TCBS agar in cholera testing served as differential diagnosis, as BA and MAC aided in identifying or ruling out other enteric pathogens with similar clinical presentations such as *E. coli*, and *Salmonella* [13, 14]. The culture plates were incubated at 35–37 °C for 18–24 h. Secondly, samples were enriched in alkaline peptone water (APW) for 4–6 h, followed by subculturing onto BA, MAC, and TCBS agar within the same incubation period [14].

On TCBS agar, presumptive *Vibrio cholerae* colonies appeared as shiny yellow colonies due to sucrose fermentation. Suspected *V. cholerae* colonies were further sub-cultured on non-selective agar-Mueller Hinton for further confirmatory tests. A series of biochemical tests, including the oxidase test, were conducted to identify *Vibrio* species [12]. Oxidase-positive colonies underwent further biochemical identification using Triple Sugar Iron (TSI) agar, Lysine Iron Agar (LIA), Sulphur Indole Motility (SIM) test, and Citrate test. A culture result was classified as positive if *Vibrio cholerae* O1 was isolated from stool samples and negative if *V. cholerae* was not detected or if non-O1/non-O139 *V. cholerae* was isolated [15].

### Antimicrobial susceptibility testing

The antibiotic resistance profile of *V. cholerae* isolates was assessed using the Kirby-Bauer disk diffusion method on Mueller-Hinton agar. This was done on 150 mm (15 cm) diameter Petri dishes for six antibiotics, following Clinical Laboratory Standards Institute (CLSI) and European Committee on Antimicrobial Susceptibility Testing (EUCAST) guidelines [14, 16, 17]. The antibiotics tested were selected based on their common use in cholera treatment and availability in the study laboratories. These included: ampicillin (10 µg), (trimethoprim/sulfamethoxazole) (1.25/23.75 µg), cefotaxime (30 µg), ceftazidime (30 µg), chloramphenicol (30 µg), ciprofloxacin (5 µg), erythromycin (15 µg), and tetracycline (30 µg). Clinical breakpoints for susceptibility testing were determined according to CLSI (M45) and EUCAST guidelines for *Vibrio cholerae* [14, 16, 17]. Due to logistical constraints, including periodic shortages of certain antibiotic discs and variations in clinical relevance over time, not all *V. cholerae* isolates were tested against the full panel of antibiotics. As a result, the number of isolates tested for each antibiotic varied. The interpretation of AST results was based on the zone of inhibition, categorized as susceptible, intermediate, or resistant [18]. Furthermore, *Vibrio cholerae* exhibiting resistance to three or more classes of antimicrobial agents will be classified as multi-drug resistant [19].

### Quality control for AST

Routine quality control (QC) measures were implemented in each batch of AST to ensure accuracy, precision, and reliability. This included the use of control strains, Escherichia coli American type culture collection (ATCC) 25,922 and Pseudomonas aeruginosa ATCC 27,853, to validate the performance of media, reagents, and equipment [16, 17]. This was performed to maintain consistency and compliance with established standards, following guidelines from CLSI [17].

### Data collection and analysis

Clinical and laboratory data were carefully reviewed for accuracy before being entered into a structured Microsoft Excel spreadsheet designed as a data collection tool. Data were extracted from the Laboratory Information System and microbiology registers to ensure completeness and reliability. To ensure consistency and validity, data were randomly checked and cross-matched by two independent reviewers. Discrepancies were resolved through discussion and re-examination of the original records.

Statistical analysis was conducted using SPSS version 26. Descriptive statistics, including medians and proportions, were used to summarize the data and determine *Vibrio cholerae* prevalence and the distribution of study covariates. Missing data were handled through list-wise deletion, and sensitivity analyses were conducted to assess the impact of missing data on the results. Chi-square tests were used to compare resistance patterns across hospitals, with a *p*-value of < 0.05 considered statistically significant.

## Results

### Prevalence of cholera in the Copperbelt Province

**Fig. 2:**
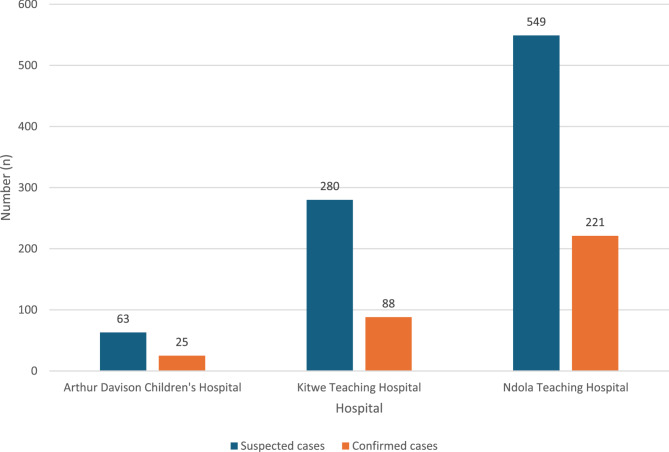
Distribution of confirmed cholera cases

During the study period, a total of 892 suspected cholera cases were reported across the three major referral hospitals in the Copperbelt Province. Of these, *Vibrio cholerae* was confirmed through culture in 334 cases, yielding an overall prevalence of 37.4%. Among the three hospitals, Ndola Teaching Hospital recorded the highest number of confirmed cases (*n* = 221, 24.8%), followed by Kitwe Teaching Hospital (*n* = 88, 9.9%), while Arthur Davison Children’s Hospital reported the lowest prevalence (*n* = 25, 2.8%) (Fig. 2). The age distribution revealed that the highest proportion of cases (*n* = 80, 26.1%) occurred among individuals aged 30–39 years, while the lowest (*n* = 14, 4.6%) was observed in those aged 70 years and above. The male-to-female ratio was 1.09:1, indicating a slightly higher number of male cases, with a median age of 31 years.

### Antimicrobial resistance patterns of *Vibrio Cholerae*

The antibiotic susceptibility testing of *Vibrio cholerae* isolates demonstrated high resistance to several commonly used antibiotics, with high resistance observed for Trimethoprim/sulfamethoxazole 69 (74.2%), Ampicillin 75 (54.3%), and imipenem 22 (46.8%) (Table 1). In contrast, effective antibiotics were observed for several antibiotics, such as Erythromycin 24 (100%), gentamicin 6 (85.7%), cefuroxime 9 (81.8%), ciprofloxacin 118 (76.6%) and tetracycline 72 (74.2%), respectively. A high intermediate was also noted in imipenem 21 (44.7%), Ampicillin 61 (44.2%), and Trimethoprim/sulfamethoxazole 21 (22.6%) (Table 1).

**Table 1:** Antimicrobial resistance patterns of *Vibrio cholerae*

Antimicrobial Agent	Disk Content (µg)	No. of Isolates Tested (*n*)	Susceptible (%)	Intermediate (%)	Resistant (%)
**High-Resistance Antibiotics**					
Ampicillin	10	138	2 (1.5)	61 (44.2)	75 (54.3%)
Trimethoprim/sulfamethoxazole	1.25/23/75	93	3 (3.2)	21 (22.6)	69 (74.2)
Imipenem	10	47	4 (8.5)	21 (44.7)	22 (46.8)
**Moderate-Resistance Antibiotics**					
Chloramphenicol	30	31	10 (32.3)	12 (38.7)	9 (29.0)
Ceftazidime	30	109	50 (45.9)	34 (31.2)	25 (22.9)
**Low-Resistance Antibiotics**					
Ciprofloxacin	5	154	118 (76.6)	21 (13.6)	15 (9.7)
Tetracycline	30	97	72 (74.2)	3 (3.1)	22 (22.7)
**High-Susceptibility Antibiotics**					
Erythromycin	15	25	25 (100.0)	0 (0.0)	0 (0.0)
Gentamicin	10	7	6 (85.7)	1 (14.3)	0 (0.0)
Cefuroxime	30	11	9 (81.8)	0 (0.0)	2 (18.2)

The number of isolates tested against each antibiotic varied due to resource constraints and changes in laboratory testing protocols. This variability should be taken into account when interpreting susceptibility rates

*µg* Microgram, *%* Percentage

### Multidrug resistance patterns

Multidrug resistance (MDR), defined as resistance to three or more antibiotic classes, was detected in 3.7% (9/246) of the isolates. The most prevalent MDR profile involved resistance to three or more antibiotics 3 (1.2%), followed by resistance to Ciprofloxacin-Ceftazidime-Tetracycline 2 (0.8%,) and the rest at 0.4% (Table 2).

**Table 2:** Multidrug resistance patterns in *Vibrio cholerae* isolates

Multidrug Resistance Profile	Frequency (*n*)	Percentage (%)
Resistance to 3 or more antibiotics	3	1.2
Ciprofloxacin-Ceftazidime-Tetracycline	2	0.8
Ampicillin-Chloramphenicol- Co-trimoxazole	1	0.4
Ampicillin-Ciprofloxacin-Co-trimoxazole	1	0.4
Ampicillin-Ciprofloxacin-Chloramphenicol	1	0.4
Ceftazidime-Chloramphenicol-Tetracycline	1	0.4
Total	**9**	**3.7**

## Discussion

This study investigated the prevalence of cholera and the antibiotic resistance patterns of *Vibrio cholerae* isolates from three major referral hospitals in Zambia’s Copperbelt Province. The findings revealed an overall cholera prevalence of 37.4%, with significant variations across hospitals. Ndola Teaching Hospital recorded the highest number of confirmed cases (24.8%), followed by Kitwe Teaching Hospital (9.9%), while Arthur Davison Children’s Hospital reported the lowest prevalence (2.8%). High levels of AMR were observed, particularly to Trimethoprim/sulfamethoxazole 69 (74.2%), Ampicillin 75 (54.3%), and imipenem 22 (46.8%). In contrast, erythromycin (100%), gentamicin (85.7%) and ciprofloxacin 118 (76.6%) remained highly effective. Alarmingly, MDR was detected in 3.7% of isolates, with resistance to four or more antibiotics being the most prevalent profile (1.2%).

The prevalence of confirmed *Vibrio cholerae* cases in this study (37.4%) is consistent with findings from similar settings. For example, Chiyangi et al. (2017), who reported a prevalence of 40.8% in Lusaka, Zambia [20], while William et al. (2020), documented a prevalence of 38% in the Democratic Republic of Congo [13]. Similarly, Bitew et al. (2024) reported a slightly lower prevalence of 30.1% in Ethiopia [14], which still falls within a comparable range. However, our prevalence was notably higher than the 20.6% reported by Kerketta et al. (2025) in Odisha, India [21]. These variations may be attributed to differences in study populations, geographical and environmental factors, and public health infrastructure. For instance, the high prevalence in the Copperbelt Province may reflect the region’s poor sanitation, overcrowding, and limited access to clean water, which facilitate cholera transmission.

The high resistance rates observed in this study align with global trends of increasing AMR in *Vibrio cholerae*. For example, Kerketta et al. (2025) reported 100% resistance to ceftazidime and imipenem in India [21], consistent with our findings of 22.9% and 46.8% resistance, respectively. Similarly, Bitew’s (2024) 56.8% to ampicillin in Ethiopia [14], mirroring our results of 54.3% resistance. These findings underscore the growing threat of AMR in cholera treatment, particularly in resource-limited settings like Zambia, where access to alternative antibiotics is often constrained.

However, there are notable differences in resistance patterns across regions. For instance in Zambia, Ethiopia, Kenya, Mozambique, and Nepal reported lower to high resistance levels to ampicillin, trimethoprim/sulfamethoxazole, erythromycin, ciprofloxacin and chloramphenicol [11, 14, 19, 20, 22], compared to our findings of 100% susceptibility to erythromycin, 76.6% susceptibility to ciprofloxacin, and 29.0% resistance to chloramphenicol. Cefuroxime (81.8%), followed by gentamicin (85.7%), suggesting their continued reliability. Ciprofloxacin and tetracycline, both commonly used in cholera treatment, maintained moderate susceptibility rates of 76.6% and 74.2%, respectively. These findings align with those of Chiyangi et al. (2017), who reported 100% susceptibility of *V. cholerae* isolates to ampicillin, cefotaxime, and gentamicin, further reinforcing their potential as effective treatment options [20]. Additionally, Chiyangi et al. found that 94.1% of strains were susceptible to tetracycline, with 5.9% showing intermediate resistance, which is slightly higher than the 74.2% susceptibility observed in our study. Furthermore, our study aligns with what was reported in a systematic review, which showed majority of the isolates being susceptible to ciprofloxacin, chloramphenicol, tetracycline and gentamycin [23]. The continued effectiveness of these antibiotics emphasizes their role in cholera treatment; however, ongoing surveillance is essential to monitor emerging resistance trends and guide appropriate therapeutic strategies [14].

The overall MDR rate of 3.7% in this study which is lower than the 6.45% reported by Gupta et al. (2016) in Nepal [19] and 64.5% documented by Shah et al. (2023) in Kenya [11]. The variation may be attributed to the availability of antibiotics in different regions and the strength of the antimicrobial stewardship in our setting. For example, the high MDR rate in Kenya may reflect the widespread use of antibiotics in both clinical and community settings, while the lower rate in Nepal may be linked to stricter antibiotic regulations and more effective public health interventions [11, 19]. In the Copperbelt Province, the low MDR rate underscores the need for enhanced antimicrobial stewardship programs to continue regulating antibiotic use and to prevent the further spread of resistant strains.

The sustained efficacy of erythromycin suggests that these antibiotics should be prioritized as first-line treatments for cholera in the Copperbelt Province. However, the presence of MDR *vibrio cholerae* strains highlights the urgent need for enhanced antimicrobial stewardship programs to regulate antibiotic use and prevent further resistance. Public health interventions should also focus on targeted immunization in endemic areas, improving WASH infrastructure to reduce cholera transmission and the spread of resistant strains.

This study has several limitations that should be acknowledged. First, the hospital-based design may not fully capture community-wide cholera cases, potentially underestimating the true burden of the disease. Second, the absence of whole-genome sequencing (WGS) limits our understanding of the genetic mechanisms driving resistance, which is critical for developing targeted interventions. A key limitation of this study lies in the antimicrobial susceptibility interpretation framework. Although the study originally employed CLSI guidelines (M45, 3rd edition, 2018) in accordance with national protocols and resource availability at the time of testing, we acknowledge the growing preference for EUCAST due to its regular updates and open access. However, re-analysis using EUCAST was not feasible for all antibiotics in this study due to differences in antimicrobial panels, limited breakpoint availability for specific organism-antibiotic combinations, and the retrospective nature of the data. This may have introduced variability in susceptibility classification. We recommend that future prospective studies adopt EUCAST where applicable to enhance standardization and international comparability. Finally, the inconsistency in the number of isolates tested against each antimicrobial agent. This variability was primarily due to logistical constraints, such as intermittent stockouts of specific antibiotic discs and changes in testing priorities over the course of sample processing. As such, not all isolates were subjected to the same antibiotic panels and the susceptibility percentages in Table 1 are derived from different denominators and should be interpreted with caution. This inconsistency may have introduced bias in the resistance patterns observed and limits the generalizability of the findings. Future studies should strive to standardize antibiotic panels to ensure comparability and stronger conclusions. Future studies should aim to standardize testing across all isolates to provide more robust comparative analysis.

Despite these limitations, this study provides the first localized data on cholera prevalence and AMR patterns in the Copperbelt Province, offering critical insights for public health interventions. Future research should focus on longitudinal studies to monitor resistance trends over time, community-based studies to capture a broader range of cases, and the use of WGS to identify resistance genes and inform targeted interventions. Furthermore, it is recommended to strengthen WASH initiatives through a comprehensive and integrated approach. This includes increasing access to safe and clean drinking water, ensuring the proper disposal of human waste and wastewater, and promoting hygiene practices that prevent the spread of diseases, such as regular handwashing with soap. Infrastructure development is also essential, with investments needed in water treatment plants, sanitation facilities, and hygiene-supportive structures. Additionally, targeted immunization of highly endemic areas is also critical, coupled with behaviour change should be encouraged through public education and community engagement to foster lasting hygienic practices. Lastly, developing and implementing supportive policies, alongside strong advocacy efforts, is crucial to prioritize and sustain WASH improvements at all levels.

## Conclusion

This study highlights the increasing prevalence of multidrug-resistant *Vibrio cholerae* in Zambia’s Copperbelt Province, posing a serious threat to effective cholera treatment. The persistence of resistance to first-line antibiotics calls for urgent updates to treatment protocols and strengthened AMR surveillance. Given Zambia’s recurrent cholera outbreaks, enhanced surveillance, antimicrobial stewardship, and improved WASH initiatives are essential for reducing the disease burden and preventing future outbreaks and targeted immunization is key to control cholera. An important limitation of this study was the inconsistency in the antimicrobial agents used across different testing laboratories. This variability was attributed to differences in local antibiotic availability and testing protocols, which resulted in not all *Vibrio cholerae* isolates being tested against the same range or number of antibiotics. This lack of standardization may affect the comparability of resistance profiles and should be considered cautiously when interpreting the findings of the study.

## Data Availability

The datasets used and/or analysed during the current study are available from the corresponding author upon reasonable request.

## Abbreviations

AMR: Antimicrobial Resistance
APW: Alkaline Peptone water
AST: Antimicrobial susceptibility testing
ATCC: American Type Culture Collection
BA: Blood agar
CLSI: Clinical Laboratory Standards Institute
EUCAST: European Committee on Antimicrobial Susceptibility Testing
LIA: Lysine Iron Agar
MAC: MacConkey agar
MDR: Multidrug resistance
SIM: Sulphur, Indole, Motility
TCBS: Thiosulfate Citrate Bile Salts Sucrose
TSI: Triple Sugar Iron
WASH: Water, Sanitation and hygiene
WGS: whole–genome sequencing
QC: Quality control
ADCH: Arthur Davison Children’s Hospital
NTH: Ndola Teaching Hospital
KTH: Kitwe Teaching Hospital
